# Supplementary material for: Modification of Phosphorylation Sites in the Yeast Lysine Methyltransferase Set5 Exerts Influences on the Mitogen-Activated Protein Kinase Hog1 under Prolonged Acetic Acid Stress
Source: Microbiol Spectr. 2023 Mar 28;11(2):e03011-22. doi: 10.1128/spectrum.03011-22 (PMC10100857; doi:10.1128/spectrum.03011-22)
Supplement: Supplemental file 1 — Supplemental material. Download spectrum.03011-22-s0001.pdf, PDF file, 0.9 MB [file spectrum.03011-22-s0001.pdf]

SUPPLEMENTAL MATERIAL for

Phosphorylation of the yeast lysine methyltransferase Set5 is associated with the differential expression and phosphorylation of the mitogen-activated protein kinase Hog1 under prolonged acetic acid stress

Pei-Liang Ye, Xue-Qing Wang, Bing Yuan, Ming-Ming Zhang, Xin-Qing Zhao*

State Key Laboratory of Microbial Metabolism, Joint International Research Laboratory of Metabolic & Developmental Sciences, School of Life Sciences and Biotechnology, Shanghai Jiao Tong University, Shanghai 200240, China.

* Corresponding author. E-mail address: xqzhao@sjtu.edu.cn

Table S1. Plasmids used in this study

Plasmids	Description	Source
Cas9-G418	p414, ARS/CEN, *KanMX*, *TEF1p*-*Sp*Cas9-*CYC1t*	Lab preservation
gRNA_clone	pRS42H, 2 μm, *hphNT*, AmpR, *SNR52p-gRNA-SUP4t*	Lab preservation
gRNA-SET5	gRNA targeting to *SET5*	This study
pGADT7	pUC, 2 μm, *GAL4* activation domain (AD)	Clontech
pGBKT7	pUC, 2 μm, *GAL4* DNA binding domain (DNA-BD)	Clontech
pGADT7-Hog1	Hog1 was fused to pGADT7	This study
pGADT7-Set5	Set5 was fused to pGADT7	This study
pGBKT7-Set5	Hog1 was fused to pGBKT7	This study
pGBKT7-Akl1	Akl1 was fused to pGBKT7	This study
pGBKT7-Atg1	Atg1 was fused to pGBKT7	This study
pGBKT7-Cmk2	Cmk2 was fused to pGBKT7	This study
pGBKT7-Rim15	Rim15 was fused to pGBKT7	This study
pGBKT7-Rtk1	Rtk1 was fused to pGBKT7	This study

Table S2. Strains used in this study

**Strains**	**Description**	**Source**
E. coli DH5α	For plasmid construction and propagation	Invitrogen Ltd
BY4741	*S. cerevisiae, haploid, MATa, his3Δ1, leu2Δ0, met15Δ0 and ura3Δ0*	Euroscarf
BY4741-SET5ΔZF	*SET5* zinc finger-deletion in BY4741	Lab preservation
BY4741-SET5FALG	"*FLAG*" label was fused to SET5 in BY4741	Laboratory stock
SPSC01	*S. cerevisiae*, diploid, self-flocculating industrial strain	Lab preservation
PLY01	*FLO1* deletion in SPSC01	Lab preservation
PLY01-Cas9	transform Cas9-G418 plasmid into PLY01	Lab preservation
PLY01-S458-A	PLY01, Set5-S458A	This study
PLY01-S458-D	PLY01, Set5-S458D	This study
PLY01-S458-3A	PLY01, Set5-S458A, S461A, S462A	This study
PLY01-S458-3D	PLY01, Set5-S458D, S461D, S462D	This study
PLY01-S458-10A	PLY01, Set5-S458A, S461A, S462A, S466A, S475A, S476A, T511A, S512A, S517A, S520A	This study
PLY01-S458-10D	PLY01, Set5-S458D, S461D, S462D, S466D, S475D, S476D, T511D, S512D, S517D, S520D	This study
AH109	MATa, trp1-901, leu2-3, 112, ura3-52, his3-200, gal4Δ, gal80Δ, LYS2::GAL1UAS-GAL1TATA-his3, MEL1 GAL2UAS- GAL2TATA-ade2, URA3::MEL1UAS-MEL1TATA-lacZ	Clontech
AH-AHog1-BSet5	transform pGADT7-Hog1 and pGBKT7-Set5 into AH109	This study
AH-ASet5-BAkl1	transform pGADT7-Set5 and pGBKT7-Akl1 into AH109	This study
AH-ASet5-BAtg1	transform pGADT7-Set5 and pGBKT7-Atg1 into AH109	This study
AH-ASet5-BCmk2	transform pGADT7-Set5 and pGBKT7-Cmk2 into AH109	This study
AH-ASet5-BRim15	transform pGADT7-Set5 and pGBKT7-Rim15 into AH109	This study
AH-ASet5-BRtk1	transform pGADT7-Set5 and pGBKT7-Rtk1 into AH109	This study
AH-AMcm2-BMcm10	Positive control in yeast two-hybrid interaction assays	Lab preservation
AH-AHog1-B	transform pGADT7-Hog1 and pGBKT7 into AH109, negative control	This study
AH-A-BSet5	transform pGADT7 and pGBKT7-Set5 into AH109, negative control	This study

Table S3. Primers used in this study

**Primers**	**Sequences (5’→3’)**
gRNA-SET5S458-F	GATCGCCATCTTCACTACTATCAT
gRNA-SET5S458-R	AAACATGATAGTAGTGAAGATGGC
Donor-SET5S458A-F	ATGCGGATGCTAATTTAGGAGTAGAAAAAATAGATGCAAATGATAGTAGTGAAGATGGC
Donor-SET5S458D-F	ATGCGGATGCTAATTTAGGAGTAGAAAAAATAGATGATAATGATAGTAGTGAAGATGGC
Donor-SET5S458-R	TCTCATGGAAGATTTACGATTACCAGTTGATTTCTTAGAGCCATCTTCACTACTATCAT
Donor-SET53A-F	ATGCGGATGCTAATTTAGGAGTAGAAAAAATAGATGCAAATGATGCAGCAGAAGATGGC
Donor-SET53D-F	ATGCGGATGCTAATTTAGGAGTAGAAAAAATAGATGATAATGATGATGATGAAGATGGC
Donor-SET53AD-R	TCTCATGGAAGATTTACGATTACCAGTTGATTTCTTAGAGCCATCTTCATCATCATCAT
Donor-SET510A*	AAAGAGTTCCAAATCTAGAGAAAAAGAATGCGGATGCTAATTTAGGAGTAGAAAAAATAGATGCAAATGATGCAGCAGAAGATGGCGCAAAGAAATCAACTGGTAATCGTAAAGCAGCAATGAGAGAGGCCCAGCCAGATTTAAAAGAAATACTGAAGAATGGAAAAGAATTTGAATTAGACATACCAGAAACTGTTGATACACAAGGGAATGTAAGAAAAGCAGCAGTCAGATTCGATGCAAACGTTGCAGTCGCAGTGGATGAAAGATAAGAGATAAACGCATACTAAATTACTGTGTCTGTTATTGTTT
Donor-SET510D*	AAAGAGTTCCAAATCTAGAGAAAAAGAATGCGGATGCTAATTTAGGAGTAGAAAAAATAGATGATAATGATGATGATGAAGATGGCGATAAGAAATCAACTGGTAATCGTAAAGATGATATGAGAGAGGCCCAGCCAGATTTAAAAGAAATACTGAAGAATGGAAAAGAATTTGAATTAGACATACCAGAAACTGTTGATACACAAGGGAATGTAAGAAAAGATGATGTCAGATTCGATGATAACGTTGATGTCGCAGTGGATGAAAGATAAGAGATAAACGCATACTAAATTACTGTGTCTGTTATTGTTT
PCR-pGADT7-F	ATCCATCGAGCTCGAGCTGC
PCR-pGADT7-R	GAATTCACTGGCCTCCATGGCC
PCR-PGBKT7-F	ATGGCCATGGAGGCCGAATT
PCR-PGBKT7-R	CAGGTCCTCCTCTGAGATCAGCTTC
PCR-pAHOG1-F	ATGGAGGCCAGTGAATTCATGACCACTAACGAGGAATTC
PCR-pAHOG1-R	AGCTCGAGCTCGATGGATTTACTGTTGGAACTCATTAGC
PCR-pBSET5-F	ATCTCAGAGGAGGACCTGATGACATTGACTATCAAGATAGGAAC
PCR-pBSET5-R	AATTCGGCCTCCATGGCCATTTATCTTTCATCCACTGCGAC
PCR-pASET5-F	ATGGAGGCCAGTGAATTCATGACATTGACTATCAAGATAGGAAC
PCR-pASET5-R	AGCTCGAGCTCGATGGATTTATCTTTCATCCACTGCGAC
PCR-pBAKL1-F	ATCTCAGAGGAGGACCTGATGTCGATCACGAATGGTAC

Table S3. (Continued) Primers used in this study

Primers	Sequences (5’→3’)
PCR-pBAKL1-R	AATTCGGCCTCCATGGCCATTCATTTCTCGCTTCTAAAGACTG
PCR-pBATG1-F	ATCTCAGAGGAGGACCTGATGGGAGACATTAAAAATAAAGATCACAC
PCR-pBATG1-R	ATTCGGCCTCCATGGCCATTTAATTTTGGTGGTTCATCTTCTGC
PCR-pBCMK2-F	ATCTCAGAGGAGGACCTGATGCCCAAGGAGTCAGAGGT
PCR-pBCMK2-R	AATTCGGCCTCCATGGCCATTTAGTCTTCTGACTTCGACTCCCGA
PCR-pBRIM15-F	ATCTCAGAGGAGGACCTGATGGCTTTGTTAGATCAATTCCCTATCC
PCR-pBRIM15-R	AATTCGGCCTCCATGGCCATTCAGTGCGTTTCATCAGAATC
PCR-pBRTK1-F	ATCTCAGAGGAGGACCTGATGGTTAAGGAAACTCCATTACAC
PCR-pBRTK1-R	AATTCGGCCTCCATGGCCATTTAATTTGAATCTTTATTCCCGTGTTG
ChIP-HSP150-F	TACGCTTCCACCTTCGGTAT
ChIP-HSP150-R	ACAGAAGCAGTAGTGGTAGC
ChIP-HOG1-F	ATTGGTGGCAGTGATGGACA
ChIP-HOG1-R	GCGCAGCCATGTTTAACTGA
ChIP-RIM15-F	CAACAGGCGATCTTGGCAAACTTC
ChIP-RIM15-R	TGGCGTCCTGACACAGCTCTC
ChIP-MGA2-F	CTCTGAGTACGACCAAAGTG
ChIP-MGA2-R	CGCTAGATCCAATATCAAGGAG
ChIP-INO80-F	GGATAATGATGAGGATGGATCTG
ChIP-INO80-R	GGGCTTCCTTAGCATTCATT
ChIP-SPT23-F	AGGTTTCGCCAATGACAAAT
ChIP-SPT23-R	CAAGCATACAGTTCGTAGAAATGA
ChIP-HFA1-F	GGCTGGGAAATCTAGTCAAG
ChIP-HFA1-R	TTAAGAAGTGGCTGTAAGGC
RT-RIM15-F	ACGACTGGTCAGACTTTTAT
RT-RIM15-R	GAACTCTTCGGGAAGTTGGA
RT-GPX1-F	GCGTTCACACCTCAATACAA
RT-GPX1-R	GGGAAGGTTACACCATATTT
RT-GPX2-F	GAATGCAAGGACAAGAAAGG
RT-GPX2-R	CTTCCAATTCTTTATACTGCGG
RT-HAA1-F	AATCCTGAAGGTGTTTGCAC
RT-HAA1-R	GCATGATATTTGCAAACCTC
RT-HOG1-F	GTCACGGAATTACAAGGAAC
RT-HOG1-R	CGCGGAGTGAACGTATTTTA
RT-MSN2-F	CGAAACTAATTTATCGCCTC
RT-MSN2-R	GGGTTAGTGTCAATGAAAAG

Table S3. (Continued)

Primers	Sequences (5’→3’)
RT-MSN4-F	GAAGCATTGAAGTTCATGCC
RT-MSN4-R	GAGTGGGTTTACTGTTGCTA
RT-PMA1-F	CAATCTAATCACGGTGTCGA
RT-PMA1-R	CTTCATCGGAAGTTAAACCG
RT-SCH9-F	CATCAGAAAATGCCCTTTTT
RT-SCH9-R	GGTATCGCCATGTACATTTC
RT-HHF1-F	AAGGTCTAGGTAAAGGTGGT
RT-HHF1-R	GCTCTGACTTCTTCGTAGAT
RT-HHF2-F	GGTGGTAAAGGTCTAGGAAA
RT-HHF2-R	GGCTCTGACTTCTTCGTAGA
RT-HTA2-F	GCTGCTAAAGCTTCTCAATC
RT-HTA2-R	CACCAGAACCAATTCTCTGG
RT-HTB2-F	AGCTGCCAAGAAAACATCAA
RT-HTB2-R	GACTTCTGGGAAATACCAGT
RT-CMK2-F	TTCTTGGTGCCGTGGAGTATATGC
RT-CMK2-R	CCGAAGTCCGCAATTACCAGAGG
RT-HAL5-F	AGCGGCGATGATGGTGACAATG
RT-HAL5-R	GTTGCTGCTGTTGCTGCTGTTG
RT-PTK2-F	GGCAGTGGTAGCGGTGGTAATTC
RT-PTK2-R	TGCAATGCGACAGAGGACTTGTG
RT-AKL1-F	CCGTCGTGATGGCGTTCAAGG
RT-AKL1-R	GCGATGGATCAAGGACACAGGAAG
RT-ATG1-F	ATCTGCCACATCTCAGTTGAGTGC
RT-ATG1-R	ACCAGTTGGACGTTACCTGCATAG
RT-RTK1-F	GAAGGAGCTTCCGGTTCAGTGTC
RT-RTK1-R	TTCGTGGTGCAGCGTGGAAC
RT-OLE1-F	CTACTACGCTGTCGGTGGTGTTTC
RT-OLE1-R	CCATTTAGCGGACCCTTCAACGG
RT-GPD1-F	ACAACGCTTCTGCTGCCATCC
RT-GPD1-R	AGCCATTAGCCTAGCAACCTTGAC
RT-CKA1-F	TGTGGTCGTTTGGGACAATGTTGG
RT-CKA1-R	AAGCTGGTCTGTGTTACTCGTTCC
RT-CTT1-F	CAGTCCAACGAACACTTGTA
RT-CTT1-R	GCAATTGCTGATAGTTGGCT
RT-RCK1-F	CACGGATGACCAAGCTCCTGTTG
RT-RCK1-R	CGAAACCAGTTTGTGGATGGCAAC

Table S3. (Continued)

Primers	Sequences (5'→3')
RT-CKA2-F	TTCCGAAGTGTTCAGCGGTAGATG
RT-CKA2-R	GGCCGCCTGTTAGATTGGTCAG
RT-CKB1-F	CGAAGACGACGTGGTGGAAGAAG
RT-CKB1-R	TCTGCTGCGTGCTCGATAATACTC
RT-CKB2-F	AAGAGGCGGACTTTGGAAGATGTC
RT-CKB2-R	CAGGAATGTCATGCAAACCAACCG
RT-STL1-F	AACTACCTGAATCGCCACGTTGG
RT-STL1-R	GCGTCGTCTAGTGTTCCTACCAAG
RT-FPS1-F	CGACGAAGGACGCTCTTCATCAC
RT-FPS1-R	CCATCGTTGCCGTTGTTGTTGTTG
RT-HSF1-F	CGGCCTGCATTTGTTAATAA
RT-HSF1-R	GGTAAAATTTGGTGCACAAA
RT-HSP12-F	GGATTCGGTGAAAAAGCTTC
RT-HSP12-R	AAGACACCCTTGTTGTCTTC

Note: *Both donor DNA of SET510A and SET510D for transformation were synthesized by Tsingke Biotechnology Co., Ltd.

Table S4. Antibodies used in this study

Specificity	Supplier	Catalog #
Anti-Actin	Abways Technology	AB0061
Anti-Set5	Affinity Biosciences	Custom-made in this study
Anti-Hog1	Affinity Biosciences	Custom-made in this study
Anti-Hog1-P	Affinity Biosciences	Custom-made in this study
Anti-Flag-Tag	Affinity Bioscience	T0053
HRP Goat Anti-Mouse IgG	BBI Life Science	D110087
HRP Goat Anti-Rabbit IgG	ABclonal Technology	AS104

Supplementary figure legends

Figure S1 Spot assay of PLY01 and its Set5 S458 mutant strains. Strain PLY01, PLY01-SET5S458-A, and PLY01-SET5S458-D were cultured on YPD agar plates with no stress or various environmental stresses.

Figure S2 Growth and fermentation performance of PLY01 and its Set5 mutation strains. (A and B) Growth and fermentation performance of PLY01 and Set5 mutation strains under no stress. (C and D) Growth and fermentation performance of PLY01 and Set5 mutation strains under 7.5 g/L acetic acid stress. Data are averages from three duplicate experiments. The error bars indicate the standard deviations.

Figure S3 Growth evaluation of yeast strains under various stresses. PLY01 and Set5 mutation strains were cultivated in 200 μL YPD medium in microtiter plates with 5.0 g/L acetic acid (A), 10 mM H_2O_2 (B), 5.0 g/L furfural (C), or 1.0 M NaCl (D). Cell growth (OD_{600}) of the yeast strains was measured using a Bioscreen microbiology reader (Bioscreen C Labsystems, Helsinki, Finland), which was recorded every 0.5 h. The lines shown are the mean of three duplicate experiments. Error bars are not shown for better comparison.

Figure S4 Transcriptional level of 31 selected genes related to stress tolerance in the presence of 7.5 g/L acetic acid. (A), comparisons between PLY01 and its Set5 mutant strains; (B) to (D), comparisons between the Set5 phosphomimetic and alanine substitution mutant strains. Data are averages from three duplicate experiments. The error bars indicate the standard deviations.

Figure S5 Enrichment of Set5 to *HOG1* and *RIM15* in *S. cerevisiae* BY4741. The yeast strain was grown till log phase, and samples were collected for ChIP assays. Asterisks represent p-value < 0.05. Data are averages from at least three duplicate experiments. The error bars indicate the standard deviations.

Figure S6 Set5 was enriched in the coding region of *HOG1* revealed by ChIP-seq analysis. The Set5 enriched region in *HOG1* in the yeast strain *S. cerevisiae* BY4741 was presented. Significant enrichment difference (-$\log_{10}$pvalue=0.683) was found in the region marked by the two yellow dotted lines (982 to 1287 bp from the start codon of *HOG1*), which was investigated in this study. Yeast cells were grown in YPD medium supplemented with 4.2 g/L acetic acid, and the cells at log phase were collected for ChIP-seq analysis following the procedure reported previously[1]. The unique mapped reads were further analyzed, reads were trimmed using trimmomatic tool58 and were then aligned to *S. cerevisiae* reference genome using bowtie2. Model-based Analysis of ChIP-Seq (MACS) was used for peak-calling[2]. Two independent biological replicates were performed, and the results showed a high degree of similarity. One representative result was shown in the figure. The x-axis shows the position in the chromosome, and the y-axis of the peak signal indicates the extent of enrichment.

Figure S7 Set5 interacts with various protein kinases. Blue dots in yeast two-hybrid assays represent positive interaction. AD, *GAL4* activation domain. BD, *GAL4* DNA binding domain. Set5 or the protein kinases were fused to AD or BD, respectively. PC, positive control. NC, negative control.

Figure S1

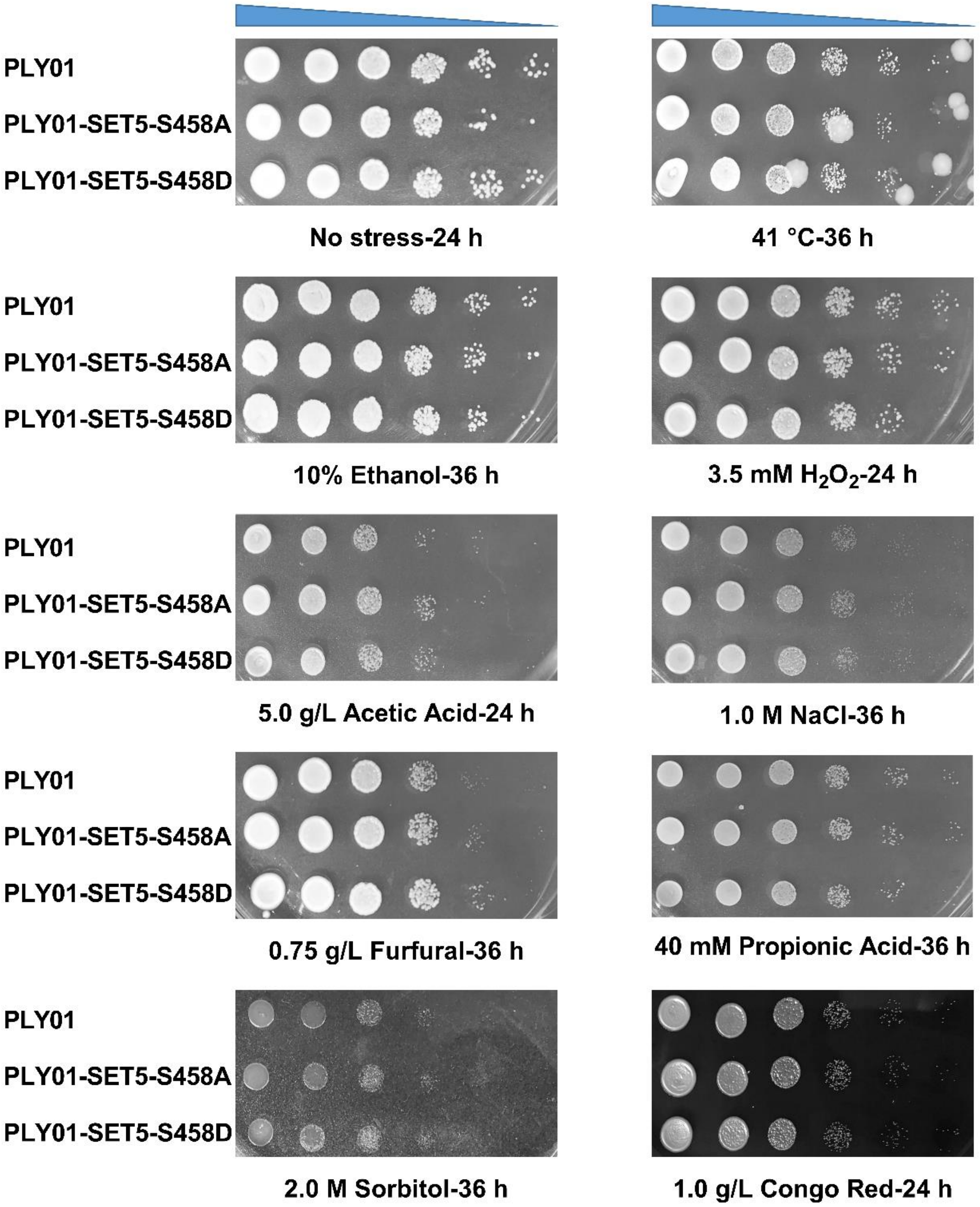
PLY01
PLY01-SET5-S458A
PLY01-SET5-S458D
No stress-24 h
41 °C-36 h
10% Ethanol-36 h
3.5 mM H_2O_2-24 h
5.0 g/L Acetic Acid-24 h
1.0 M NaCl-36 h
0.75 g/L Furfural-36 h
40 mM Propionic Acid-36 h
2.0 M Sorbitol-36 h
1.0 g/L Congo Red-24 h

74 **Figure S2**

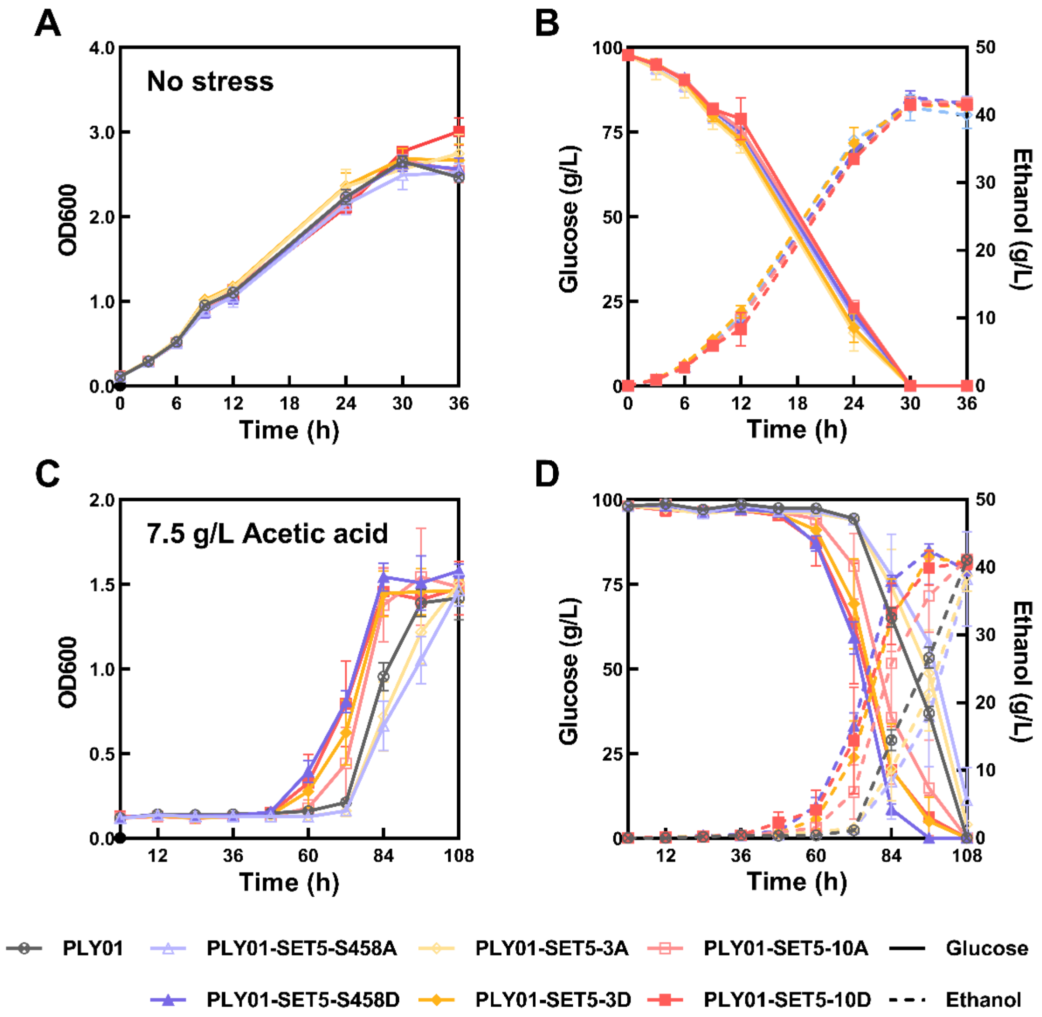

75

Figure S3

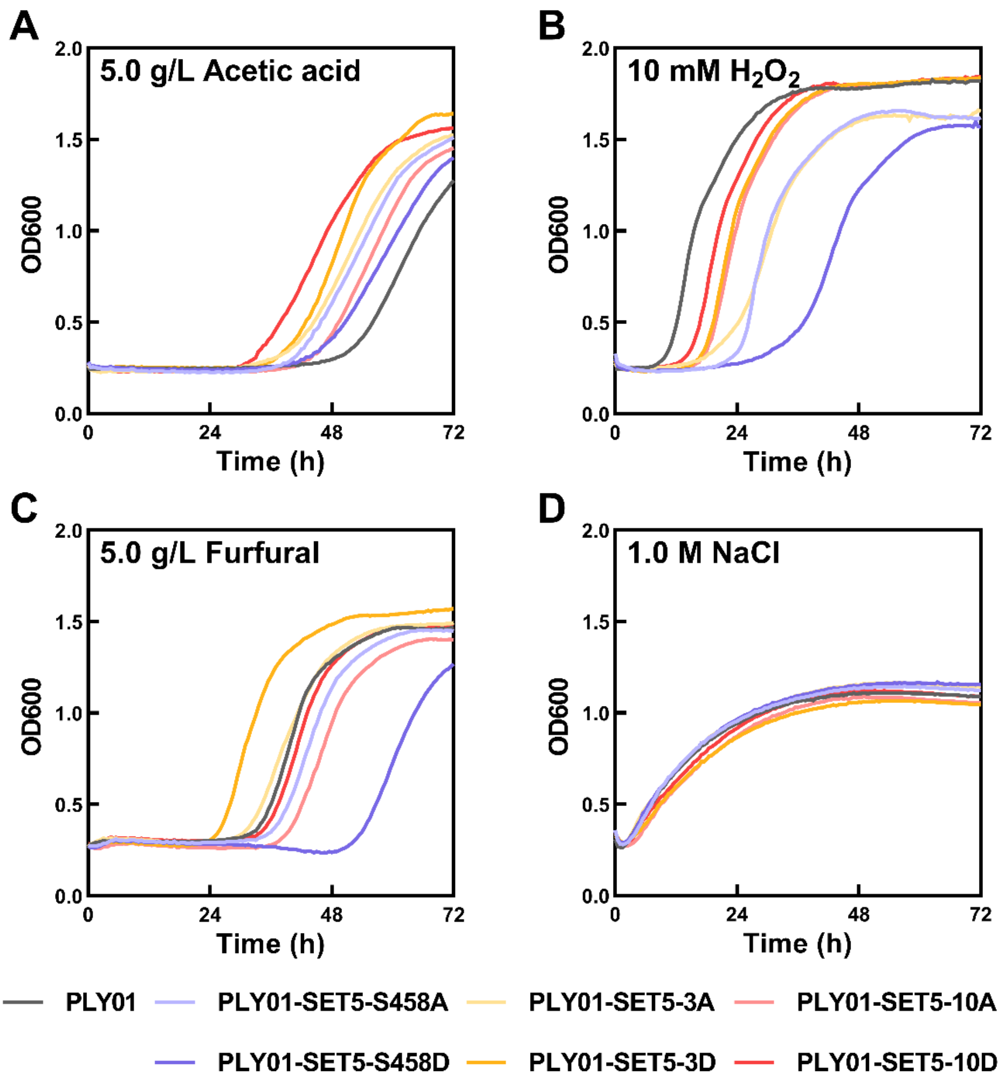
A
5.0 g/L Acetic acid
B
10 mM H_2O_2
C
5.0 g/L Furfural
D
1.0 M NaCl
OD600
Time (h)
0.0
0.5
1.0
1.5
2.0
0
24
48
72
PLY01
PLY01-SET5-S458A
PLY01-SET5-3A
PLY01-SET5-10A
PLY01-SET5-S458D
PLY01-SET5-3D
PLY01-SET5-10D

Figure S4

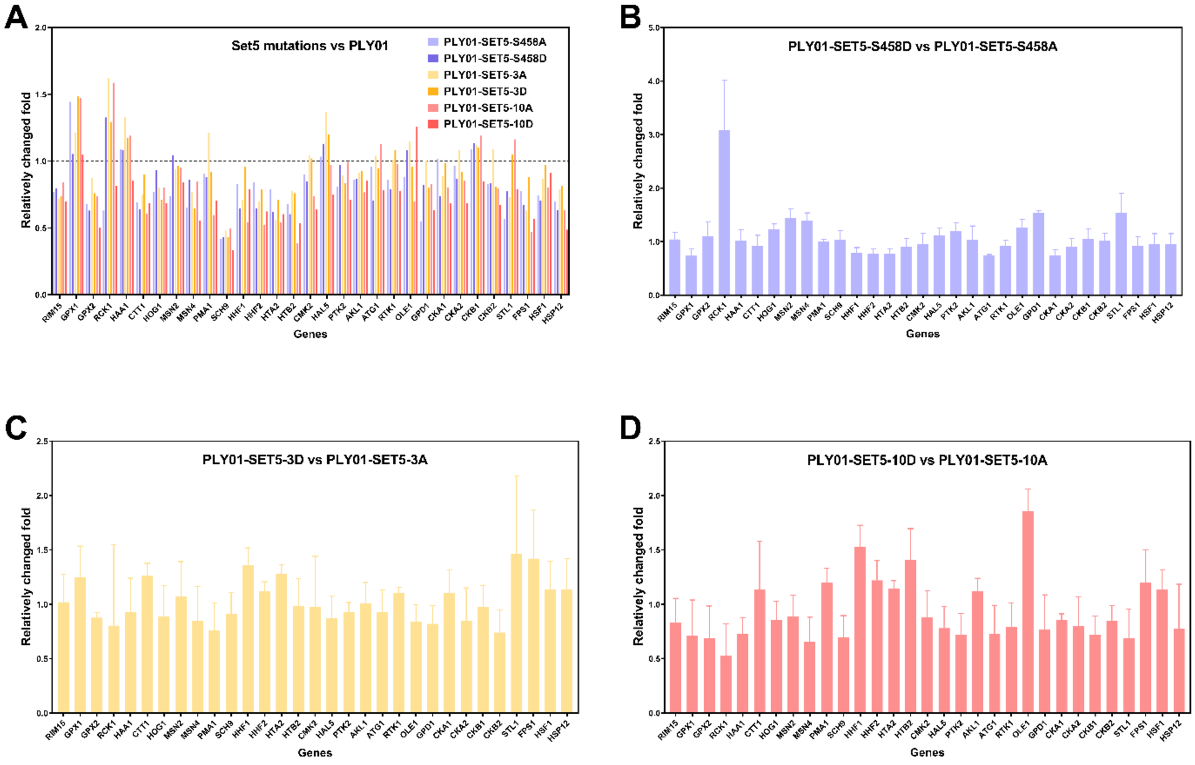
A
Set5 mutations vs PLY01
PLY01-SET5-S458A
PLY01-SET5-S458D
PLY01-SET5-3A
PLY01-SET5-3D
PLY01-SET5-10A
PLY01-SET5-10D
Relatively changed fold
Genes
B
PLY01-SET5-S458D vs PLY01-SET5-S458A
Relatively changed fold
Genes
C
PLY01-SET5-3D vs PLY01-SET5-3A
Relatively changed fold
Genes
D
PLY01-SET5-10D vs PLY01-SET5-10A
Relatively changed fold
Genes

Figure S5

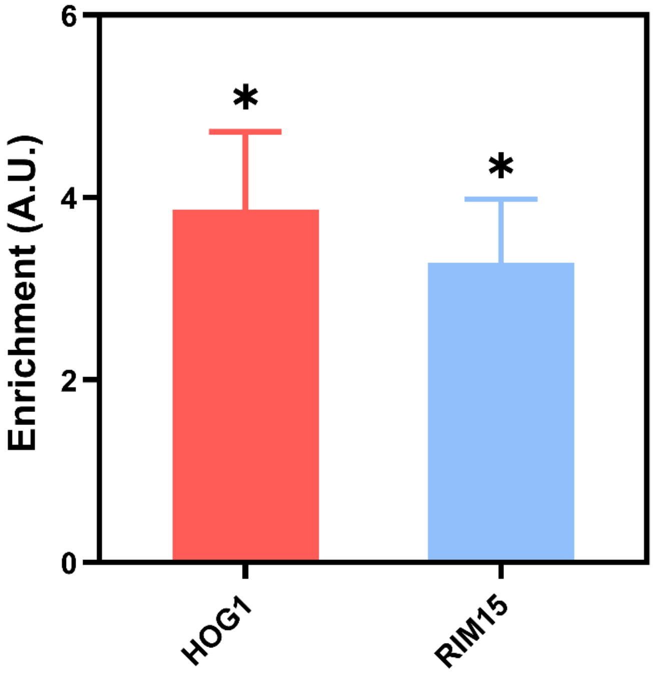

Figure S6

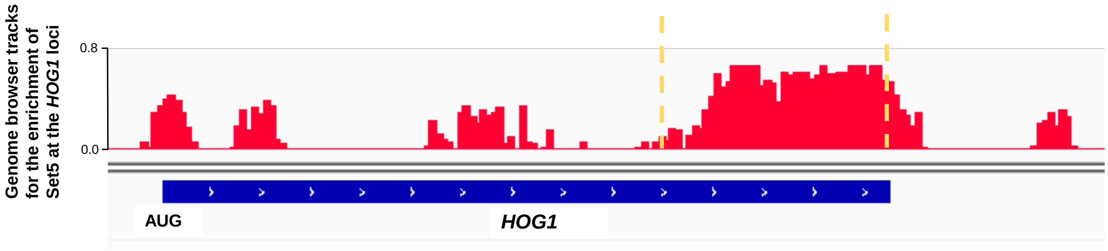
Genome browser tracks for the enrichment of Set5 at the *HOG1* loci
0.8
0.0
AUG
HOG1

Figure S7

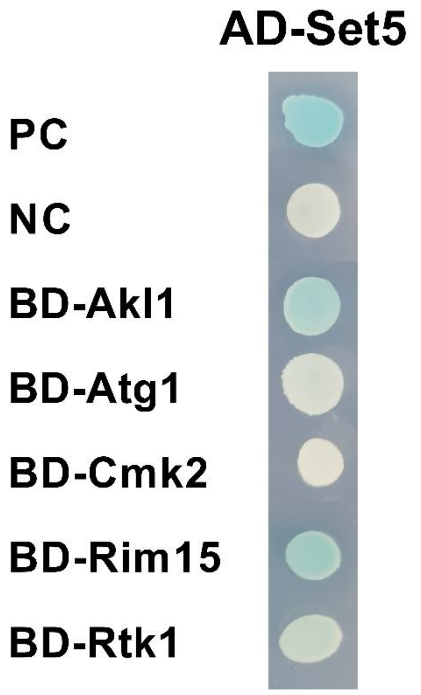

Supplementary references

[1] He MH, Liu JC, Lu YS, Wu ZJ, Liu YY, Wu Z, Peng J, Zhou JQ. 2019. KEOPS complex promotes homologous recombination via DNA resection. Nucleic Acids Res 47:5684-5697

[2] Zhang Y, Liu T, Meyer CA, Eeckhoute J, Johnson DS, Bernstein BE, Nusbaum C, Myers RM, Brown M, Li W, Liu XS. 2008. Model-based Analysis of ChIP-Seq (MACS). Genome Biol 9 (9):R137.
